# Performance evaluation of ozonation for removal of antibiotic-resistant *Escherichia coli* and *Pseudomonas aeruginosa* and genes from hospital wastewater

**DOI:** 10.1038/s41598-021-04254-z

**Published:** 2021-12-31

**Authors:** Farzaneh Baghal Asghari, Mohammad Hadi Dehghani, Reza Dehghanzadeh, Davoud Farajzadeh, Dariush Shanehbandi, Amir Hossein Mahvi, Kamyar Yaghmaeian, Akbar Rajabi

**Affiliations:** 1 Department of Environmental Health Engineering, School of Public Health, Tehran University of Medical Sciences, Tehran, Iran; 2 Institute for Environmental Research, Center for Solid Waste Research, Tehran University of Medical Sciences, Tehran, Iran; 3 Institute for Environmental Research, Center for Water Quality Research, Tehran University of Medical Sciences, Tehran, Iran; 4 Health and Environment Research Center, Tabriz University of Medical Sciences, Tabriz, Iran; 5 Department of Cellular and Molecular Biology, Faculty of Biological Sciences, Azarbaijan Shahid Madani University, Tabriz, Iran; 6 Immunology Research Center, Tabriz University of Medical Sciences, Tabriz, Iran

**Keywords:** Environmental sciences, Chemistry

## Abstract

The performance of ozonation for the removal of antibiotic-resistant bacteria (ARB) and antibiotic resistance genes (ARGs) using *Escherichia coli* and *Pseudomonas aeruginosa* carrying ARGs from hospital wastewaters was evaluated in this study. Bacterial inactivation was determined using plate count methods and real time PCR for ARG damage (*Sul1, bla*_*tem*_*, bla*_*ctx*_*, bla*_*vim*_* and qnrS*). The reduction rate of bacterial cells and ARGs was increased by different amounts of transferred ozone dose from 11 to 45 mg/L. The concentration of 10^8^ cfu/ml bacteria was reduced  to an acceptable level by ozone treatment after a 5 min contact time,  Although the removal rate was much higher for concentrations of 10^6^ cfu/ml and 10^4^ cfu/ml bacteria. Overall, the tendency of gene reduction by ozonation from more to less was *16S rRNA* > *sul1* > *bla*_*tem*_ > *bla*_*ctx*_ > *qnrS* > *bla*_*vim*_. Given that plasmid-borne ARGs can potentially be transferred to other bacteria even after the disinfection process, our results can provide important insights into the fate of ARGs during hospital wastewater ozonation.

## Introduction

Hospital wastewaters are identified as sources of nutrients, inorganic and organic pollutants and are suspected to be among the main anthropogenic sources of antibiotics, ARB (Antibiotic-resistant bacteria) and ARGs (Antibiotic resistance genes) spread into the environment. Indeed, antibiotic residues are almost enough to kill susceptible bacteria and at the same time increase the number of resistant bacteria^1–5^. Antibiotics are one of the important drugs for treating infectious diseases that are released into hospital wastewaters^6–8^. Many of these bacteria harbor acquired antibiotic resistance genes and are potential carriers for the dissemination of these genes in the environmental microbiome^9,10^. Antibiotic-resistant bacteria can survive in hospital wastewaters, so conventional wastewater treatment has unacceptable results in terms of ARB and ARGs removal and might even increase the concentration of certain ARGs^11,12^. Antibiotic resistance genes may be intrinsic or acquired. Intrinsic resistance is created in an organism whose innate chromosomal makeup predictably specifies resistance, whereas acquired resistance is due to the mutation in its genetic composition^12^.

In recent years, the application of new disinfection technologies, such as advanced chemical oxidation processes (AOPs) (e.g., ozonation and homogeneous and heterogeneous photocatalysis), has been paid attention for the inactivation of ARBs and the removal of ARGs in hospital wastewaters^13,14^. Ozonation processes have been used in hospital wastewater treatment since the 1950s, and numerous studies have shown that ozone treatment is efficient in killing many types of microorganisms, such as *S. aureus*, *Streptococci spp.*, *Escherichia coli*, *Enterococcus faecali* and *Pseudomonas aeruginosa*^15,16^. Ozone is a gas molecule and allotrope of oxygen and reacts with organic and inorganic compounds due to its high oxidation potential and reactivity^17,18^. Ozonation is an effective process to remove organic pollutants and is used to reduce or inactivate pathogens by producing highly reactive radicals. Leakage of substances from the cell wall due to cell wall degradation and damage to nucleic acids and bonds of carbon–nitrogen of proteins are among the mechanisms of ozone disinfection that types of organism, contact time and radicals concentration affect this mechanism^7,19–23^. Ozone selectively reacts with organic compounds by reacting directly with ozone molecules, or indirectly with hydroxyl radicals (OHº) under alkaline pH conditions, resulting from the decomposition of ozone in hospital wastewater. The hydroxyl radical is considered a very strong non-selective oxidant that its reaction with most organic pollutants occurs either by hydrogen abstraction with saturated aliphatic hydrocarbons and alcohols, or electrophilic addition with unsaturated hydrocarbons^13,15^.

The reason for the selection of *Escherichia coli* and *Pseudomonas aeruginosa* for this study was the significant ability of these bacteria to survive on the hospital surface, the increase in antimicrobial resistance against pathogens and according to the report of the World Health Organization based on the serious threat of these bacteria to human health ^24^. Considering that ARGs such as *bla*_*tem,*_* bla*_*ctx*_*, bla*_*vim*_*, Sul1* and *qnrs* have been found in hospital wastewaters worldwide^22,25^ and given that these antibiotic genes are frequently suitable tools to show the reduction efficiencies of the different treatment steps, these genes were selected^26^. Additionally, since the imipenem ARG (*bla*_*vim*_) is reported among *Pseudomonas aeruginosa* and the spread of *bla*_*vim*_ in pathogenic bacteria is apparent, this gene was also studied^19,27^.

Given that it has been suggested based on the results of various studies that conventional treatment may be ineffective and chlorination was shown to increase the prevalence of antibiotic resistance^28,29^, methods including advanced treatment systems need to be evaluated to find a more effective and economic way to remove ARB and ARGs from hospital wastewaters^30^. Since according to several studies published, ozonation is effective in inactivating ARB and removing ARGs and in eliminating the potential regrowth of bacteria under optimal conditions, ozone doses and contact times, thus this method was considered to fill the gaps in knowledge about the effect of ozonation on ARB inactivation and reduction of ARGs.

Therefore, in this research, the aim of the work was to investigate and optimize the efficiency of ozonation for the removal of ARB and ARGs during treatment. Since the evaluation of antibiotic resistance in the population of natural wastewater has been studied, examining the resistance of bacteria to different groups of antibiotics and the resistance of bacteria to different concentrations of antibiotics have also been examined separately, showing the difference between this study and other studies. Additionally, regarding ARGs, it should be noted that the presence of resistance genes was examined both before exposure to antibiotics and after exposure of identified bacteria to different concentrations of antibiotics. The reason for doing this was to consider all the factors in selecting bacteria for ozone treatment and to select the most resistant bacteria in the hospital wastewater for treatment.

## Materials and method

### Wastewater sample collection and bacterial culture

Untreated wastewater samples were obtained from the last manhole of sewerage collecting wastewater from Tabriz University-Affiliated Hospital in Iran. Samples were taken twice during 2019 to obtain a more representative sample with a 24 h composite that was taken each period. Prior to each treatment, the pH, BOD and COD values of the samples were determined. pH values were in the neutral range (7–8), and the average BOD and COD in hospital wastewater samples were 190 ± 35 mg L^−1^ and 350 ± 67 mg L^−1^, respectively. In the first stage using the culture method and with the use of eosin methylene blue (EMB) agar and cetrimide agar, bacteria were identified for this purpose for the isolation of *Escherichia coli* and *Pseudomonas spp*. Samples were placed on agar plates in triplicate and then incubated for *Escherichia coli 24* h and for *Pseudomonas spp.* 24–48 h at 37 °C. After identification of bacteria, different antibiotics, β-lactams (cefotaxime (Ctx), ceftriaxone (Cro), ceftazidime (Caz), cefixime (Cef), cefazolin (Cfz) imipenem (Ipm)), sulfamethoxazole (Smx) and ciprofloxacin (Cip), that were purchased from Sigma Chemical Company (Sigma Aldrich, Uk) at concentrations of 8–128 µg/mL were used to determine bacterial resistance. Bacteria grew in different concentrations of antibiotics, but considering that bacteria grown at concentration of 32 µg/mL of antibiotics compared to higher concentrations of antibiotics, identified and counted obviously, this concentration of antibiotic was studied and the number of bacteria grown in this concentration is presented.

### PCR assays

#### PCR assays for confirmation of the identified bacteria and determination of antibiotic resistance genes

Since polymerase chain reaction (PCR) and quantitative PCR (qPCR) are very sensitive for detecting total DNA present in a sample, including DNA from live and dead cells^19^ and due to process-related DNA changes, PCR-based approaches allow the direct identification of DNA damage during disinfection^31^. After obtaining and purifying the plasmids that were extracted using a Plasmid DNA Miniprep Kit (TsingKe, Beijing, China), the concentration and the quality of the plasmids were determined by a NanoDropND-2000c spectrophotometer (Thermo Scientific, Wilmington, USA), and then isolated bacteria were confirmed by amplification of the 16S rRNA gene. PCR products were analyzed for DNA sequencing, and DNA sequences were analyzed by BLAST (Basic Local Alignment Search Tool) algorithms and databases from the National Center for Biotechnology (www.ncbi.nlm.nih.gov). After determining the identified bacteria, using standard PCR, the occurrence of genes resistant to β-lactams (*bla*_tem,_ *bla*_ctx_, *bla*_vim_), fluoroquinolones (*qnr*S) and sulfonamides (*sul*1) was tested using the primers presented in SI Table 1. For this, the temperature program consisted of initial denaturing at 95 °C, followed by 37 cycles of 60 s at 94 °C, 30 s at the annealing temperature, 30 s at 72 °C and a final extension step for 5 min at 72 °C.

### Determination of resistant bacteria for ozone treatment

After identifying the typical *Escherichia coli* and *Pseudomonas aeruginosa* bacteria from specific culture media, to confirm the identified colonies, the isolated bacteria were accurately identified by PCR and based on the BLAST results, five types of *Pseudomonas aeruginosa* and two types of *Escherichia coli* were confirmed, and all *Pseudomonas aeruginosa and Escherichia coli* were exposed to different concentrations of antibiotics (8–128 µg/ml) and examined for the presence of antibiotic resistance genes. Gene resistance to β-lactams (*bla*_*tem*_*, bla*_*ctx*_*, bla*_*vim*_), fluoroquinolones (*qnrS*) and sulfonamides (SulI) was tested using standard PCR. Finally, bacteria that were more resistant to antibiotics and had more antibiotic-resistant genes were studied for the treatment process.

### Quantitative PCR

After the determination of the antibiotic resistance genes of identified *Escherichia coli* and *Pseudomonades aeruginosa*, quantitative real-time PCR (qRT-PCR) was utilized for quantification of the resistance genes in the identified bacteria. Finally, bacteria with the highest number of resistance genes were selected for the ozone disinfection process. Positive ARGs, including *Sul1, bla*_*tem*_*, bla*_*ctx*_*, bla*_*vim*_*, and qnrS,* were selected for qPCR. *16S rRNA* was also employed as housekeeping gene. Plasmids carrying these target genes were prepared and used to build standard curves. qPCR reactions were performed in a LightCycler® 96 Instrument (Roche Life Science, Germany) in a 10 µL reaction mixture of 2 × SYBR Green PCR Pre-Mix (Ampliqon, Denmark), 0.2 µL of each primer, 1 µL of template DNA and 8.6μL ddH_2_O to a total volume of 20 μL. For this, a 10 min preheating step at 94 °C to activate the polymerase enzyme and denature the template DNA was considered. Then, amplification was carried out by 45 cycles of 94 °C for 10 s, annealing temperature for 30 s, and 72 °C for 20 s. The annealing temperature of each primer pair is presented in SI Table 1.

For generation of standard curves for *Escherichia coli and Pseudomonas aeruginosa* bacteria as well as for resistance genes, plasmid constructs containing 247-bp fragment of *bla*_*tem*_, the 175-bp fragment of *bla*_*ctx*_, the 382-bp fragment of *bla*_*vim*_, the 163-bp fragment of *sul1* and the 118-bp fragment of *qnrS* were used which is presented in SI Figs. 1–7 and method of preparing the standard curves is explained in the SI 1.

#### Propidium monoazide (PMA) treatment

In this research, propidium monoazide was used to separate the surviving bacterial population from inactivated bacteria during ozone treatment. Because of loss of integrity in dead or injured bacterial membranes, PMA can enter them and intercalate with intracellular and extracellular DNA. Presence of the PMA-DNA complex blocks polymerase activity at the target sites, and consequently, no PCR product is generated^32^. Therefore, PMA treatment in qPCR analyses is an important step for accurate detection and quantification of ARGs^31^. For this treatment, after filtration of the samples, (0.2-μm Supor®-200 membrane, 47 mm diameter, Pall Life Science) filters were submerged in 300 μL of a 25 μM propidium monoazide solution and put into a 1.5 mL colorless tube. After incubation in the dark at 4 °C for 5 min, samples were exposed to the LED light of the photoactivation system at 100% intensity for 15 min. After PMA treatment, samples were prepared for DNA extraction^31^.

### Analysis of ARB concentrations

The decrease in the number of viable *Escherichia coli* and *Pseudomonas aeruginosa* colonies following ozonation was measured using a culture-based technique. Because identified colonies were resistant to all antibiotics at a concentration of 32 µg/ml, selection plates were dosed with antibiotics at a concentration of 32 µg/mL. Samples were then serially diluted with phosphate buffer to achieve an approximate range of 30 to 300 colonies on the plate. A total of 0.1 mL of diluted sample was then placed into the agar plates in triplicate and incubated, finally were counted and calculated based on the number of dilutions.

### Analysis of antibiotic resistance genes

*Pseudomonas aeruginosa* harbors a number of intrinsic clinically relevant ARGs and hosts a high abundance of acquired ARGs. Specific primers targeting the ecfX gene, due to its higher amplification specificity and performance in qPCR, were used to detect the abundance of *Pseudomonas aeruginosa* in this study before and after ozone treatment^33,34^. Additionally, a specific primer pair targeting the yccT gene was used to detect the abundance of *Eschershia coli*. After ozone treatment. For this purpose, 100 mL of the wastewater was filtered through a 0.2-μm Supor®-200 membrane (47 mm diameter, Pall Life Science) and Total DNA was extracted directly from the membranes.

### Ozone treatment setup

**Figure 1:**
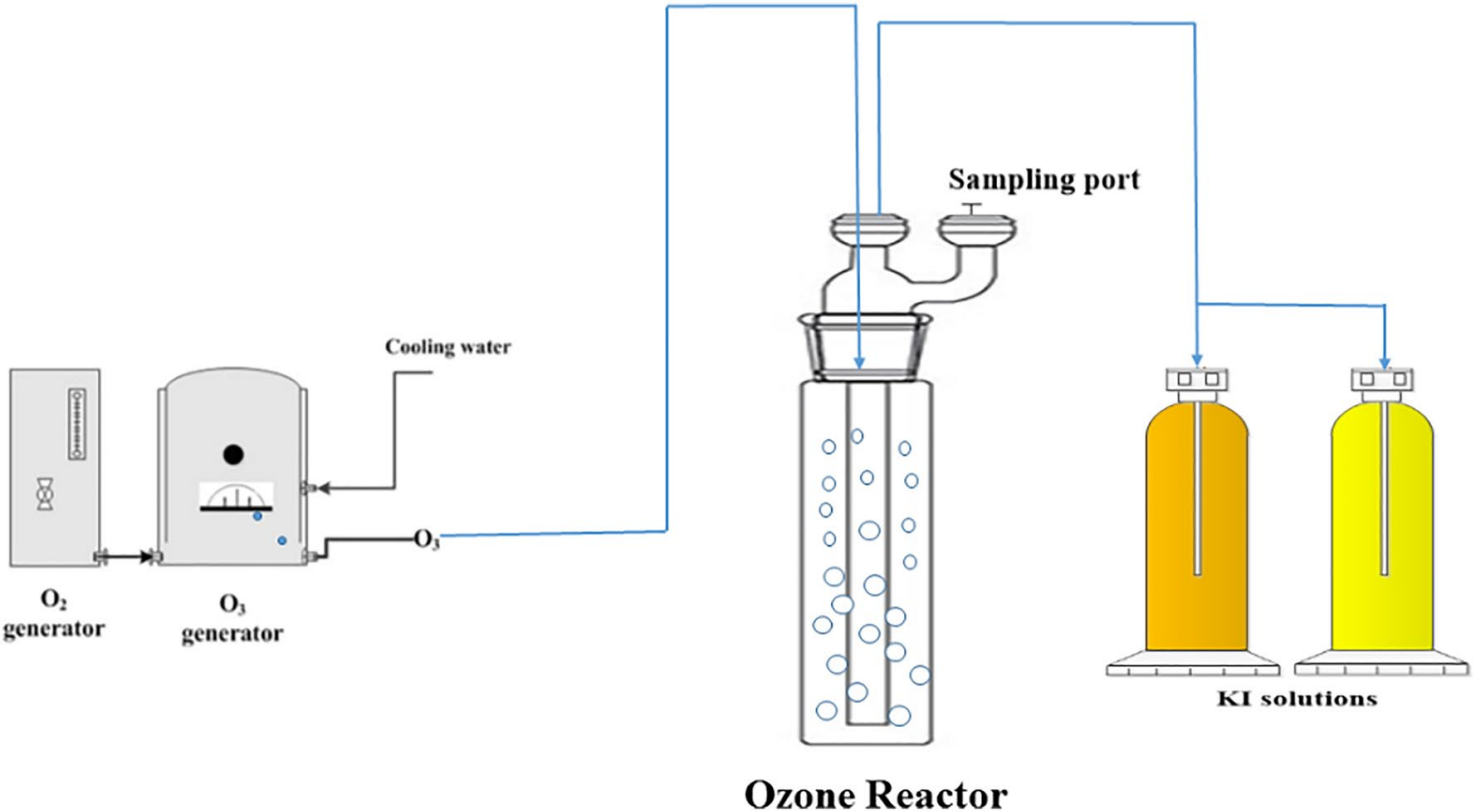
Schematic diagram of the ozonation.

All experiments were done in a pilot plant in batch mode at ambient temperature (20 ± 2 °C) at pH 7.2–7.5. Figure 1 displays a schematic diagram of the ozonation setup. The pilot system consisted of glass columns with an inner diameter of 5 cm and a total height of 50 cm. Ozone was produced using a laboratory ozone generator (Jahad Research Center, Iran) fed by an oxygen generator (Type CFS-1, New Life Elite oxygen concentrator, AirSep corporation company, USA). Ozone gas was bubbled at constant flow rate of 0.2 L min^−1^ into the wastewater through a diffuser located at the bottom of the columns, and the inlet concentration of ozone was approximately 22 mg/L throughout all ozonation experiments. The off-gas was absorbed by a 2% KI solution. Due to the specific concentration of ozone at specified intervals, wastewater samples were taken from the reactor. All experiments were conducted in triplicate, and the averaged data are presented. The amount of ozone required to bacterial destruction was calculated by the dose of ozone transferred (TOD) during the ozonation. The absorbed mass of ozone transferred into the unit volume of liquid that reacts with the oxidizable compounds is defined as TOD and was calculated by Eq. (1)^29,35,36^.

1 $${\text{TOD (mg L}}^{{ - 1}} {)} = \int\limits_{{0}}^{{\text{t}}} {\frac{{{\text{Q}}_{{{\text{gas}}}} }}{{{\text{V}}_{{{\text{liquid}}}} }}} \times (C_{{O_{3} }}^{in} - C_{{O_{3} }}^{out} {\text{) dt}}$$

In Eq. (1), Q_gas_ represents the gas flow rate (0.2 L min^−1^), V liquid is the volume of liquid in the reactor and C_O3_^in^ C_O3_^out^ are the ozone concentrations in the inlet and outlet gases, respectively.

Iodometric method was used for measurement of ozone concentration in the inlet and outlet gases^37^ to do this, two impingers were used in series and each filled with 200 ml of 2% KI solution and gas was bubbled. Ozone-exposed KI solution was added to a solution of 10 ml of H_2_SO_4_ (2 N) and titrated with Na_2_S_2_O_3_ (0.005 N) solution until it turned pale yellow. Finally by adding of 1% starch indicator solution of 0.5 mL, titration continued until the blue color changed to colorless. Spectrophotometric method was used for determination of ozone concentration in aqueous solution that for this purpose, 5 ml of the sample was transferred to a test tube containing 5 ml of 2% KI solution and after approximately 30 min using cells with a 20 mm light path, the intensity of absorption was read^36,38^.

#### Ozone for the inactivation of ARB and the removal of ARGs

In this research, the abundance of ARB (*P. aeruginosa and Escherichia coli*) and antibiotic resistance genes in these bacteria (sul1, *bla*_*tem*_*, bla*_*ctx*_*, bla*_*vim*_, and *qnrS*) in hospital wastewater exposed to different concentrations of ozone (TOD1 = 11 mg/l, TOD2 = 25 mg/l, TOD3 = 37 mg/l, TOD4 = 45 mg/l) was evaluated. This amount of TODs that were examined is based on the amount of dissolved ozone of 1–4 mg/l. Ozone utilization efficiency (OUE) in ozonation is presented in SI Table 2 (Supplementary Materials).

##### Measurement of antibiotic-resistant bacteria at different concentrations of ozone

The decrease in the number of viable *Escherichia coli* and *Pseudomonas aeruginosa* colonies from the fresh cultures of these bacteria was carried out by the culture method. For this, concentrations of 10^4^, 10^6^ and 10^8^ cfu/ml of bacteria were prepared according to McFarland standards, and then quantification and confirmation tests of culturable bacteria were performed. Samples were inoculated into the reactor at the start of the experiments and at different concentrations of ozone. Bacterial removal was expressed as a function of TOD values, and for this, the reduction of cultivable *Escherichia coli* and *Pseudomonas aeruginosa* bacteria with identified TOD at different times was investigated. It should be mentioned that bacterial-free wastewater was sterilized in an autoclave. To ensure the absence of bacteria in sterilized wastewater, the presence of bacteria was examined by the culture method. For each sample, triplicate plates were prepared, and the results were averaged.

##### Measurement of antibiotic resistance genes at different concentrations of ozone

For this purpose, DNA-free hospital wastewater was sterilized for one hour at 121 °C and 1 bar pressure. To ensure the absence of genes in sterilized wastewater, 16S rRNA bacteria were examined by PCR. At each stage of ozonation with different levels of TOD, a specific amount of DNA prepared with concentrations of 10^4^, 10^6^ and 10^8^ cfu/ml bacteria was inoculated into the reactor. The amount and reduction of antibiotic resistance genes before ozonation and at different levels of TOD were examined by extracting DNA and qPCR.

### Statistical analysis

The degree of log removal of specific genes was calculated using the following formula:

2 $${\text{j}} = {\text{log }}\left( {{\text{C}}_{0}^{{\text{j}}} /{\text{C}}_{{\text{i}}}^{{\text{j}}} } \right)$$

In this equation, j indicates specific genes (antibiotic resistance genes studied and 16S rRNA; C_0_^j^ indicates the gene copy number of specific gene j in the original wastewater samples (copies per milliliter), and C_i_^j^ indicates the gene copy number of specific gene j that survived the ozonation process at a dosage of i (copies per milliliter). To compare the inactivation efficiency of bacteria and genes responding to different ozone doses, one-way ANOVA was conducted. The difference was considered statistically significant at a p.value less than 0.05. All statistical analyses were performed using R software.

## Results and discussion

### Culture method and PCR assays for the detection of bacteria and resistance genes

In this study, all bacteria grown on EMB and cetrimide agar without antibiotics were confirmed by PCR and amplification of the 16S rRNA gene region. Based on BLAST, *Escherichia coli* and *Pseudomonas aeruginosa* were identified. Then, the resistance of the identified bacteria to different concentrations of antibiotics (8–128 μg/mL) was examined, and the presence of genes encoding antibiotic resistance determinants in all isolated bacteria was assessed by PCR. Eventually, bacteria that were more resistant to antibiotics and had more antibiotic resistance genes were studied for the treatment process, and the two bacteria determined with BLAST results are presented in Table 1.

**Table 1:** Sequence-based identification of hospital wastewater bacteria by BLAST.

Closet match in GenBank	Identity (%)	Accession no	Resistant gene				
			bla_ctx_	bla_tem_	bla_vim_	Sul	qnrs
Pseudomonas aeruginosa	99.43%	MN700178.1	+	+	+	+	+
Escherichia coli	97.02%	MN094132.1		+		+	+

Identified bacteria designated for ozone treatment were resistant to different classes of antibiotics, such as the resistant bacteria identified in similar studies^7,39^.

### Inactivation of ARB by ozonation

**Figure 2:**
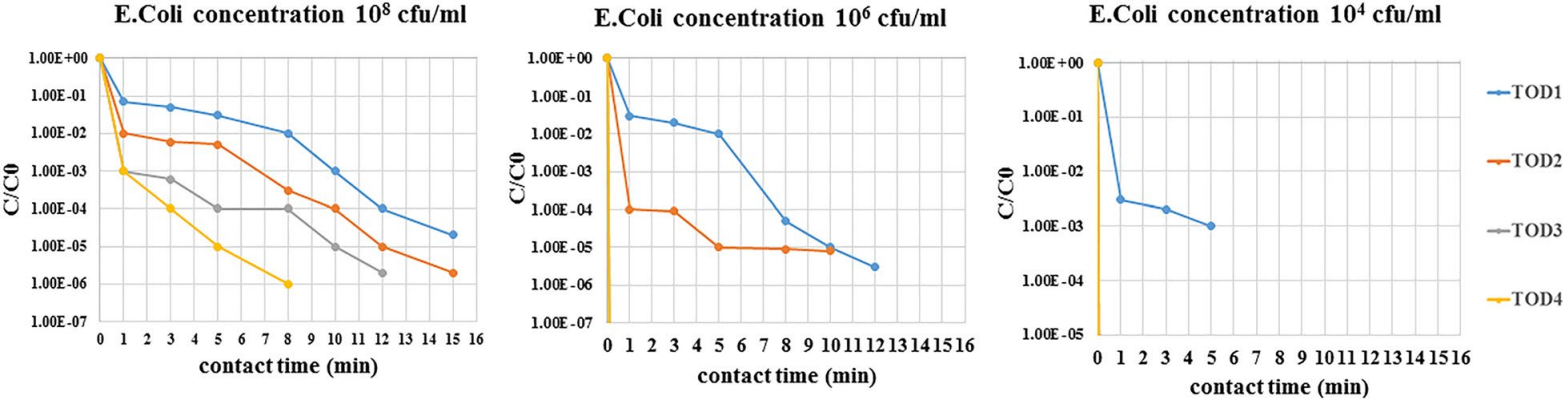
Removal efficiencies of antibiotic-resistant *Escherichia coli* by ozonation at ozone doses of 11–45 mg/l (TOD1 = 11 mg/l, TOD2 = 25 mg/l, TOD3 = 37 mg/l, TOD4 = 45 mg/l).

**Figure 3:**
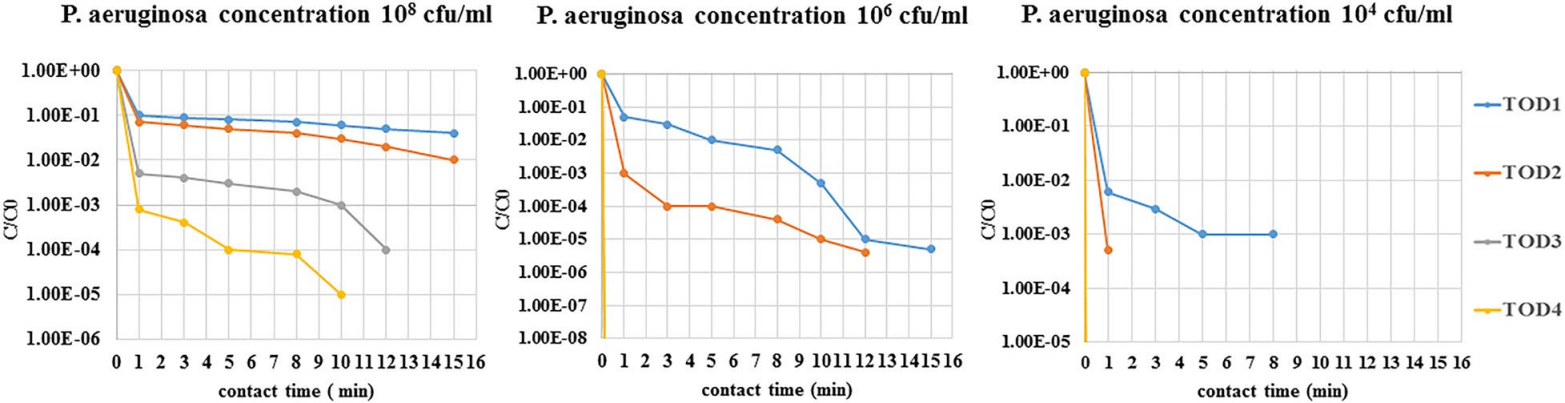
Removal efficiencies of antibiotic-resistant *Pseudomonas aeruginosa* by ozonation at ozone doses of 11–45 mg/l (TOD1 = 11 mg/l, TOD2 = 25 mg/l, TOD3 = 37 mg/l, TOD4 = 45 mg/l).

Two identified bacteria were selected *(Pseudomonas aeruginosa and Escherichia coli)* having the highest resistance to most antibiotics and having the highest resistance genes to antibiotics. Three different initial concentrations of selected bacteria were treated with four different concentrations of ozone. Figures 2 and 3 show the effect of ozone disinfection on the reduction of ARB.

Ozone reacts with different compounds, such as amines, amino acids, activated aromatic compounds, reduced sulfur residues, unsaturated bonds of phospholipids, proteins, peptidoglycan and liposaccharides available in the cell envelope, and all of these substances are abundant in hospital wastewater^40^.

In this research, a concentration of 10^8^ cfu/ml *Escherichia coli* was reduced by ozone treatment after a 5 min contact time (almost one order of magnitude in TOD1, two orders of magnitude in TOD2, four orders of magnitude in TOD3, five orders of magnitude in TOD4), while the removal rate was much higher for concentrations of 10^6^ cfu/ml and 10^4^ cfu/ml bacteria. Therefore, when the bacterial concentration was 10^6^ cfu/ml, bacteria did not survive in TOD3 and TOD4 and were reduced by ozone treatment after a 5 min contact time (almost two orders of magnitude in TOD1 and five orders of magnitude in TOD2). When the *Escherichia coli* concentration was 10^4^ cfu/ml, only in TOD1 and during the 5 min contact time survived and could be identified. (Reduced to 3 logs in TOD1, ozone dose = 11 mg/l). The same was true of *Pseudomonas aeruginosa,* and as the initial concentration of bacteria decreased (concentrations of 10^4^ cfu/ml and 10^6^ cfu/ml compared to 10^8^ cfu/ml bacteria), inactivation of bacteria required a lower dose of ozone and less contact time, and this issue has been proven in various studies^19,41^. In general, the reduction in *Pseudomonas aeruginosa* was less than that in *Escherichia coli,* and *Pseudomonas aeruginosa* was more resistant than *Escherichia coli* and inactivated 2 log of TOD3 (ozone dose = 37 mg/l*).* This result was consistent with the results of other studies^42–44^.

The present study shows that ozonation of hospital wastewater is effective in the elimination of both ARB (*Escherichia coli* and *Pseudomonas aeruginosa*. Various studies have also shown that ozone has a better effect on killing bacteria and^45^ based on studies on the effect of ozone on multidrug resistant bacteria, even low concentrations of ozone have been shown to eliminate an acceptable percentage of these bacteria^19,46^.

The rate of *Escherichia coli* reduction was higher in the same conditions than *Pseudomonas aeruginosa*, but According to the statistical results and by considering equal concentrations of initial bacteria )10^8^ cfu/ml(, only there was significant relationship between the reduction of *Escherichia coli* and *Pseudomonas aeruginosa* in TOD1 and TOD4 (p < 0.05).

### Inactivation of ARGs by ozonation

**Figure 4:**
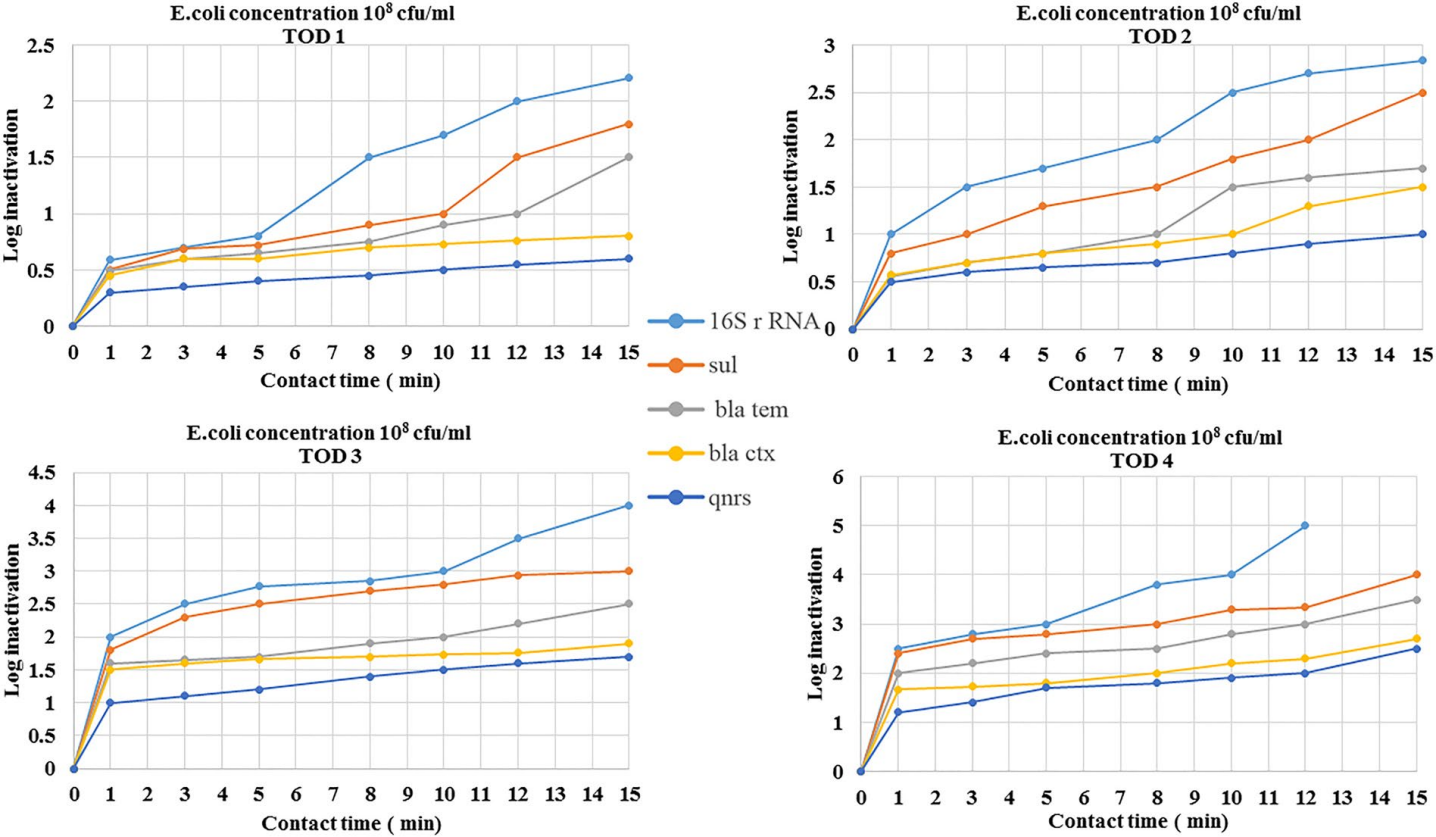
Removal efficiencies of antibiotic resistance genes in *Escherichia coli* by ozonation at ozone doses of 11–45 mg/l (TOD1 = 11 mg/l, TOD2 = 25 mg/l, TOD3 = 37 mg/l, TOD4 = 45 mg/l).

**Figure 5:**
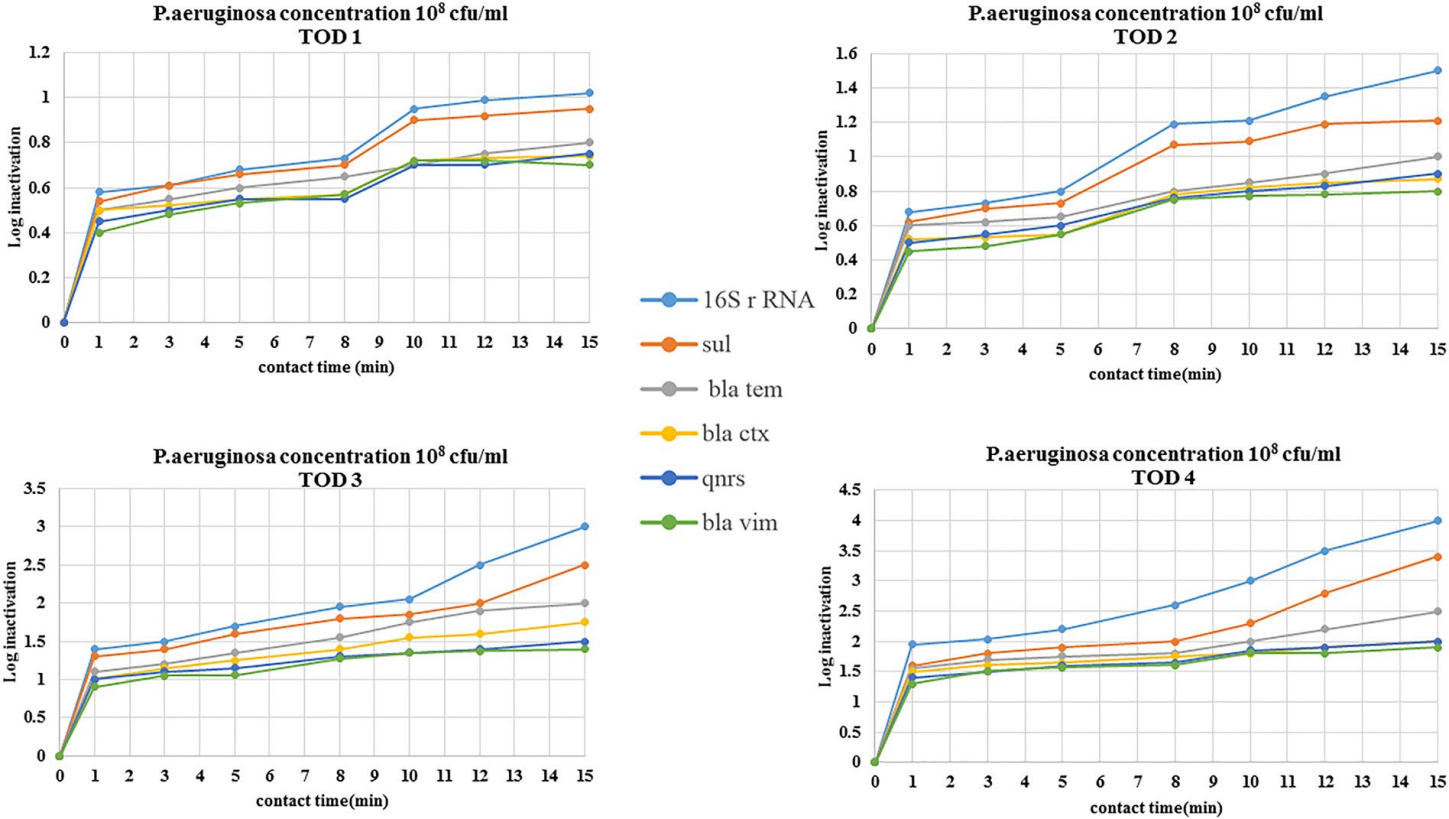
Removal efficiencies of antibiotic resistance genes in *Pseudomonas aeruginosa* by ozonation at ozone doses of 11–45 mg/l (TOD1 = 11 mg/l, TOD2 = 25 mg/l, TOD3 = 37 mg/l, TOD4 = 45 mg/l).

The removal efficiencies of antibiotic resistance genes in the ozonation process were evaluated via the reduction of gene copy numbers. The removal efficiencies of antibiotic resistance genes in *Escherichia coli and Pseudomonas aeruginosa* (10^8^ cfu/ml concentration) by ozonation are shown in Figs. 4 and 5.

The inactivation of ARGs by O_3_ depends on different influencing factors, such as the genome composition, ARG type, reaction time, concentration of microbial communities, ARB type, O_3_ dosage and wastewater parameters^18^. Ozonation results showed less removal of ARGs than 16S rRNA. This result can be attributed to the consumption of ozone by organic matter in wastewater and is similar to the results of studies conducted in this field^47^. Inactivation of selected genes increased when the ozone concentration was increased from 11 to 45 mg/L.

In this study, in accordance with other studies, O_3_ could acceptably reduce 16S rRNA, so that at TOD3 = 37 mg/l with a 5 min contact time, acceptable log decrease in 16S rRNA in both bacteria (*Escherichia coli and Pseudomonas aeruginosa)* was obtained^45,48^.

The results of the reduction of resistance genes by ozonation considering the concentration of genes in initial bacteria) 10^8^ cfu/ml (, was similar to the reduction rates of *qnrS, bla*_*tem*_* and bla*_*vim*_ being different from that of Sul1, which was acceptably reduced. Bacteria carrying the resistance genes *bla*_*vim*_ and *qnrS* showed more resistance against ozone treatment.

According to the statistical results, there was significant difference in the rate of gene reduction in *Escherichia coli* and *Pseudomonas aeruginosa* with different concentrations of initial bacteria. For example according to the results of one-way ANOVA, reduction of *sul1* genes in both bacteria with a concentration of 10^8^ cfu/ml was significantly different from the concentration of 10^4^ cfu/ml. Also, gene reduction was significantly different between concentration of 10^6^ cfu/ml and 10^4^ cfu/ml of bacteria (p < 0.05).

In this study, with decreasing concentration of initial bacteria and consequently decreasing concentration of initial genes, the rate of gene reduction by ozonation increased so that the rate of reduction of genes with 10^4^ cfu/ml initial bacteria was greater than the rate of reduction with 10^6^ cfu/ml initial bacteria. The results of the decrease in the studied genes show that ARG damage requires a greater ozone dose or longer time than inactivation of the 16S rRNA gene. These results indicated that the damage to ARGs might require more energy than inactivation of the 16S rRNA gene, which is consistent with other studies conducted in this field^23,45^.

In this study, when the amount of ozone increases, the rate of reduction of genes also increases. One of the reasons for this is that when ozone exposure increases, protein leakage and membrane permeabilization also increase, and ozone can react too rapidly with the cell envelope and damage ARGs, which is consistent with the results of studies conducted in this field^29,49^. The results of this research, which was in agreement with previous studies, found that sulfonamide genes in *Escherichia coli* and *Pseudomonas aeruginosa* were removed more than other genes by ozonation, and the rate of reduction of the *bla*_*ctx*_ and *qnrS* genes in *Pseudomonas aeroginosa* was very similar. At high concentrations of ozone, the removal rate of *bla*_*ctx*_ was slightly greater than that of *qnrS* genes. In this study, the *bla*_*ctx*_ gene was more difficult to remove than the *bla*_*tem*_ gene. One of the reasons for this may be that the origin of the CTX enzymes is different from TEM. TEM is generated by amino acid substitutions of their parent enzymes, but CTX is acquired by horizontal gene transfer from other bacteria using genetic apparatuses such as conjugative plasmids or transposons^50,51^.

The imipenem (*bla*_*vim*_) resistant gene in *Pseudomonas aeruginosa* showed more resistance after ozonation and was eliminated less than other genes^19,23^. In research conducted by Alexander et al., which checked the efficiency of ozonation treatment to inactivate selected ARGs, the results showed that the rate of reduction genes removed through ozonation for *bla*_*vim*_ was the lowest of all genes^52^. Hence, *Pseudomonas aeruginosa* and its antibiotic resistance gene directed to imipenem are important parameters for future microbiological risk assessments.

Overall, the inactivation of genes by ozone disinfection from easy to hard was *16S rRNA* > *sul1* > *bla*_*em*_ > *bla*_*ctx*_ > *qnrS* > *bla*_*vim*_. 16S rRNA, compared to other genes, was removed most efficiently, which can be attributed to its high original copies, which existed in most background bacteria. The results of this study on gene reduction are consistent with related studies^53,54^. According to the statistical results (one-way ANOVA) of different genes, significant difference was observed between the reductions of genes in different concentrations of ozone (p < 0.05).

## Conclusions

In this research, multidrug-resistant *Pseudomonas aeruginosa* and *Escherichia coli* were detected in hospital wastewater, and it was also found that hospital wastewater contains ARGs that are of great clinical importance due to their resistance to different groups of antibiotics. Given that different studies are needed to provide an understanding of the ozonation effect on the removal of antibiotic resistance determinants, optimization of the ozone process (e.g., ozone dose and contact time), bacterial species and related ARGs and physicochemical properties of hospital wastewater are important factors. The findings of this study demonstrated that ozonation of hospital wastewater constitutes a promising treatment technology for the elimination of *Escherichia coli* and *Pseudomonas aeruginosa* and selected ARGs.

Because hospital wastewaters contain ARGs and ARB and given that hospital wastewaters in most countries without any restrictions and without any pretreatment are discharged into municipal sewage systems, the spread of ARB and ARGs can become a threat to human health and ecosystems. Since routine treatment can only partially remove ARB and ARGs from hospital wastewaters, so this wastewaters need advanced treatment than what is done in municipal WWTPs and must be investigated in a more useful and economic way to remove ARB and ARGs. As a result, modifications or process combinations are needed to increase the removal rate of ARB and ARGs. Additionally, other methods, including ozone catalysts, need to be investigated to find a more useful and economic way to remove ARGs from hospital wastewaters.

Since ozonation could decrease 16S rRNA more than ARGs and despite acceptable reduction of genes by ozonation, complete removal was not achieved and the genes were present even in TOD4 (45 mg/L) with a contact time of 15 min, So to achieve completely ARG removal the dosage of ozone must be much higher than that defined by common usage and more comprehensive studies should be done in this regard.

## Supplementary Information

Supplementary Information.
