# Supplementary material for: Performance evaluation of ozonation for removal of antibiotic-resistant Escherichia coli and Pseudomonas aeruginosa and genes from hospital wastewater
Source: Sci Rep. 2021 Dec 31;11:24519. doi: 10.1038/s41598-021-04254-z (PMC8720092; doi:10.1038/s41598-021-04254-z)
Supplement: Supplementary file 1 — Supplementary Information. [file 41598_2021_4254_MOESM1_ESM.docx]

***Supplementary Materials***

**Performance evaluation of ozonation for removal of antibiotic-resistant *Escherichia coli* and *Pseudomonas aeruginosa* and genes from hospital wastewater**

Farzaneh Baghal Asghari ^1^, Mohammad Hadi Dehghani *^1, 2, 3^, Reza Dehghanzadeh **^4^, Davoud Farajzadeh ^5^, Dariush Shanehbandi ^6^, Amir Hossein Mahvi ^1,2^, Kamyar Yaghmaeian ^1^, Akbar Rajabi ^4^

^1^ Department of Environmental Health Engineering, School of Public Health, Tehran University of Medical Sciences, Tehran, Iran

^2^ Institute for Environmental Research, Center for Solid Waste Research, Tehran University of Medical Sciences, Tehran, Iran

^3^ Institute for Environmental Research, Center for Water Quality Research, Tehran University of Medical Sciences, Tehran, Iran

^4^ Health and Environment Research Center, Tabriz University of Medical Sciences, Tabriz, Iran.

^5^ Department of Cellular and Molecular Biology, Faculty of Biological Sciences, Azarbaijan Shahid Madani University, Tabriz, Iran

^6^ Immunology Research Center, Tabriz University of Medical Sciences, Tabriz, Iran

***Corresponding authors:**

**Mohammad Hadi Dehghani**: Department of Environmental Health Engineering, School of Public Health, Tehran University of Medical Sciences. Tel: 42933227,Fax: 88950188; E-mail: hdehghani@tums.ac.ir

**Reza dehghanzadeh**: Department of Environmental Health Engineering, Tabriz

University of Medical Sciences, Golgasht St., Azadi Ave., Tabriz, Iran; Tel: +98 9144184167; Fax: +98

41 33344731; E-mail: [dehghanzadehr@tbzmed.ac.ir](mailto:dehghanzadehr@tbzmed.ac.ir)

| **References** | **qPCR annealing tem (°C)** | **Traditional PCR annealing tem (°C)** | | | **product size (bp)** | **Primer Sequence (5' to 3')** | | | | **gene** | |
| --- | --- | --- | --- | --- | --- | --- | --- | --- | --- | --- | --- |
| **^1^** | 54°C | 54°C | | | **97 bp** | CAAAKACTGACGCTSAGGTG  GGCACAACCTBCAARTCG | | | | **16S rRNA** | |
| **^2^** | 57°C | 53°C | | | **175bp** | TGGTGAYVTGGMTBAARGGCA  TGGGTRAARTARGTVACCAGAA | | | | ***bla_ctx_*** | |
| **^3,4^** | 59°C | 58°C | | | **247 bp** | GCKGCCAACTTACTTCTGACAACG  CTTTATCCGCCTCCATCCAGTCTA | | | | ***bla_tem_*** | |
| **^5^** | 53°C | 60°C | | | **382 bp** | GTTTGGTCGCATATCGCAAC  AATGCGCAGCACCAGGATAG | | | | ***bla_vim_*** | |
| **^6^** | 64°C | 62°C | | | **163bp** | CGCACCGGAAACATCGCTGCAC  TGAAGTTCCGCCGCAAGGCTCG | | | | ***Sul1*** | |
| **^7^** | 62°C | 60°C | | | **118pb** | GACGTGCTAACTTGCGTGAT  TGGCATTGTTGGAAACTTGC | | | | ***qnrS*** | |
| **^8^** | 56°C | 56°C | | | **59bp** | yccT F GCATCGTGACCACCTTGA  yccT R CAGCGTGGTGGCAAAA | | | | ***Escherichia coli*** | |
| **^8^** | 56°C | 56°C | | | **81 bp** | ecfX AGCGTTCGTCCTGCACAAGT  ecfX TCCACCATGCTCAGGGAGAT | | | | *P.****aeruginosa*** | |
|  |  | |  |  | | | |  |  | |  |
|  |  | |  |  | | |  |  |  | |  |

**SI Table 1: Sequences of primers and thermal condition used in PCR amplifications**

**SI.1 Standard curves**

For generation of standard curves for *Escherichia coli and Pseudomonas* *aeruginosa* bacteria as well as for resistant genes, plasmid constructs containing 247-bp fragment of *bla_tem_*, the 175-bp fragment of *bla_ctx_*, the 382-bp fragment of *bla_vim_* ,the 163-bp fragment of *sul1* and the 118-bp fragment of *qnrS* were used which is explained in the appendix.

Conventional PCR with the primers described in Table 1, yielded the expected band sizes and then the concentration of the PCR products were quantified by a NanoDropND-2000c spectrophotometer (Thermo Scientific, Wilmington, USA), and to calculate the number of construct gene copies (GC), the following formula was used:

Number of copies=$\frac{(amount of DNA[ng]*6,022*1023)}{(average size of genome (bp)*109*650)}$ (1)

Serial decimal dilutions of this stock were made in nuclease free water to prepare the standard curves and the CT values obtained by qPCR were plotted against log gene copy number ^8^.

All standard curves plotted using at least 6 points. In the negative control, nuclease free water was added to the qPCR reaction mixture as a replacement for DNA. qPCR efficiency of target genes ranged from 96 to 100 % with R^2^ values of more than 0.98 for all standard curves. The standard curves are presented in the appendix in Figures 1-7.

## Calibration curves

### *E. coli*– *yccT*


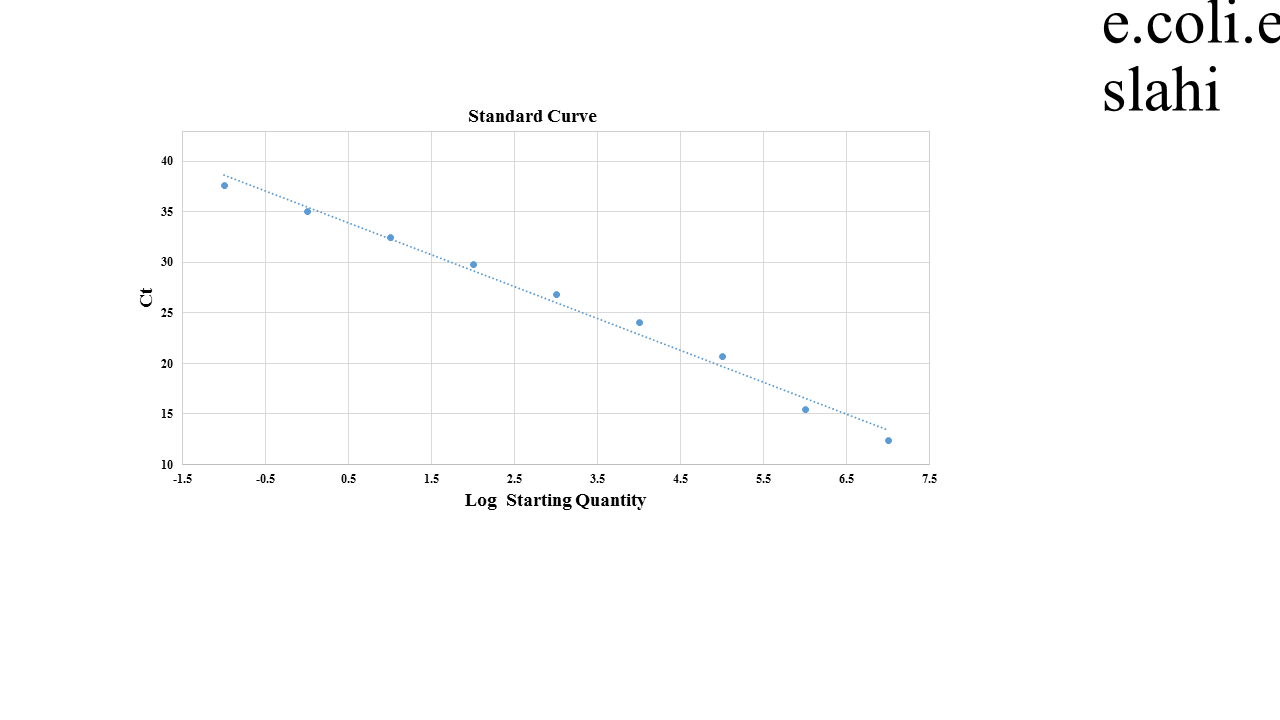


**SI Figure 1**: The slope S= -3.14. and efficiency E=96.6% was determined with a correlation coefficient

of R²=0.9883

### *Pseudomonas aeruginosa- ecfX*


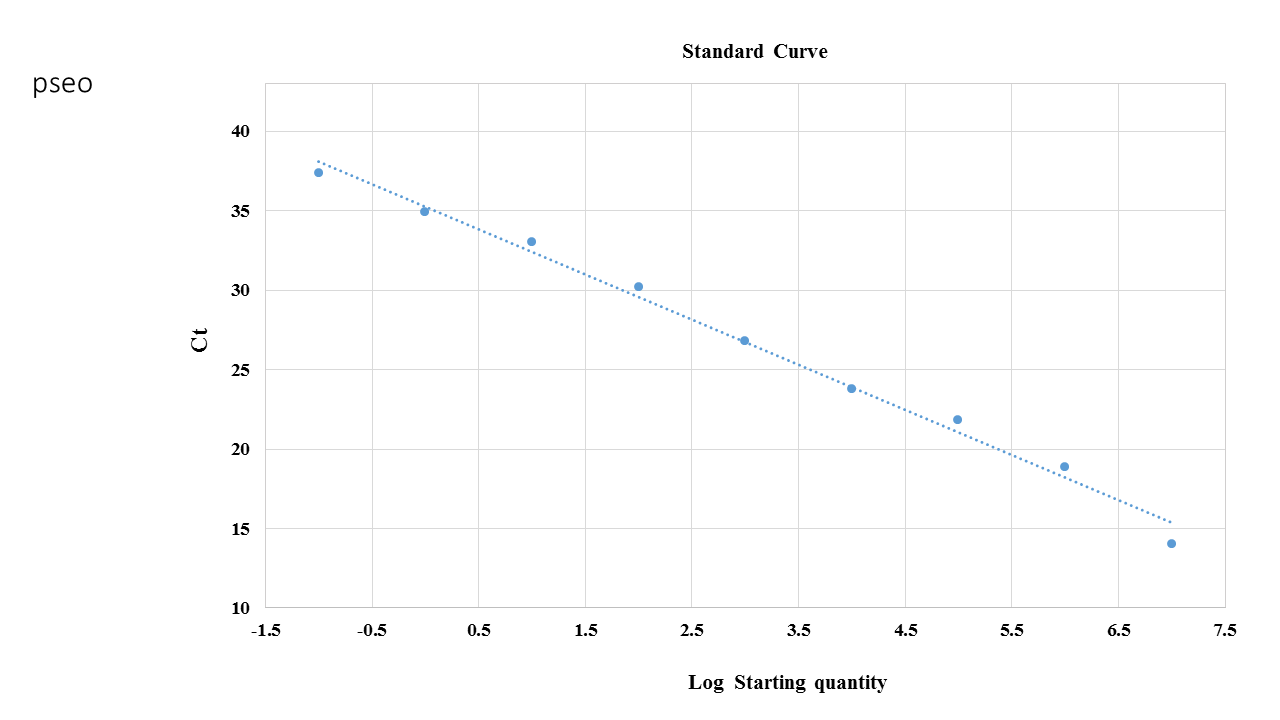


**SI Figure 2**: The slope S= -3.39 and efficiency E=97.16% was determined with a correlation coefficient

of R²=0.9911

### *E. coli (yccT)* *Sul*

**
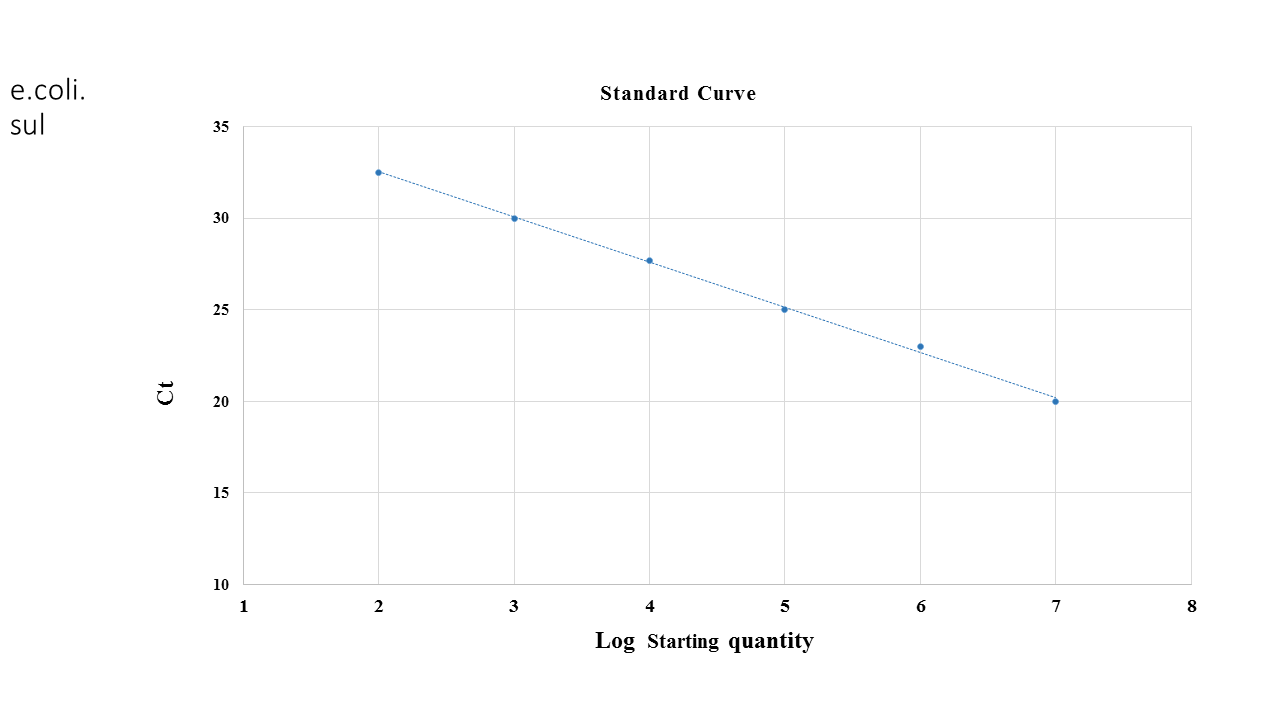
**

**Log gene copy number**

**SI Figure 3**: The slope S=-2.46 and efficiency E=96.76% was determined with a correlation coefficient

of R²=0.9983

### *E. coli*( *yccT)* *qnrS*

**
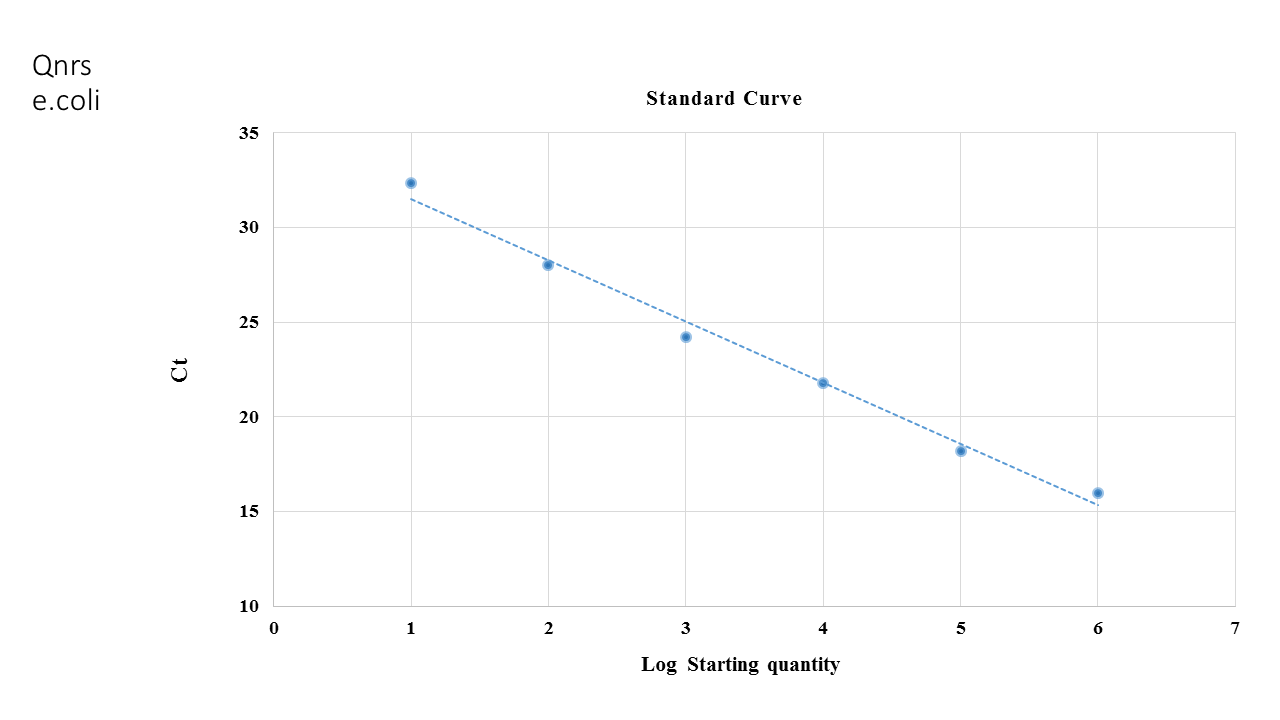
**

**Log gene copy number**

**SI Figure 4**: The slope S= -3.24 and efficiency E=96.8% was determined with a correlation coefficient

of R²=0.9891.

***E. coli*( *yccT)* *bla_ctx_***


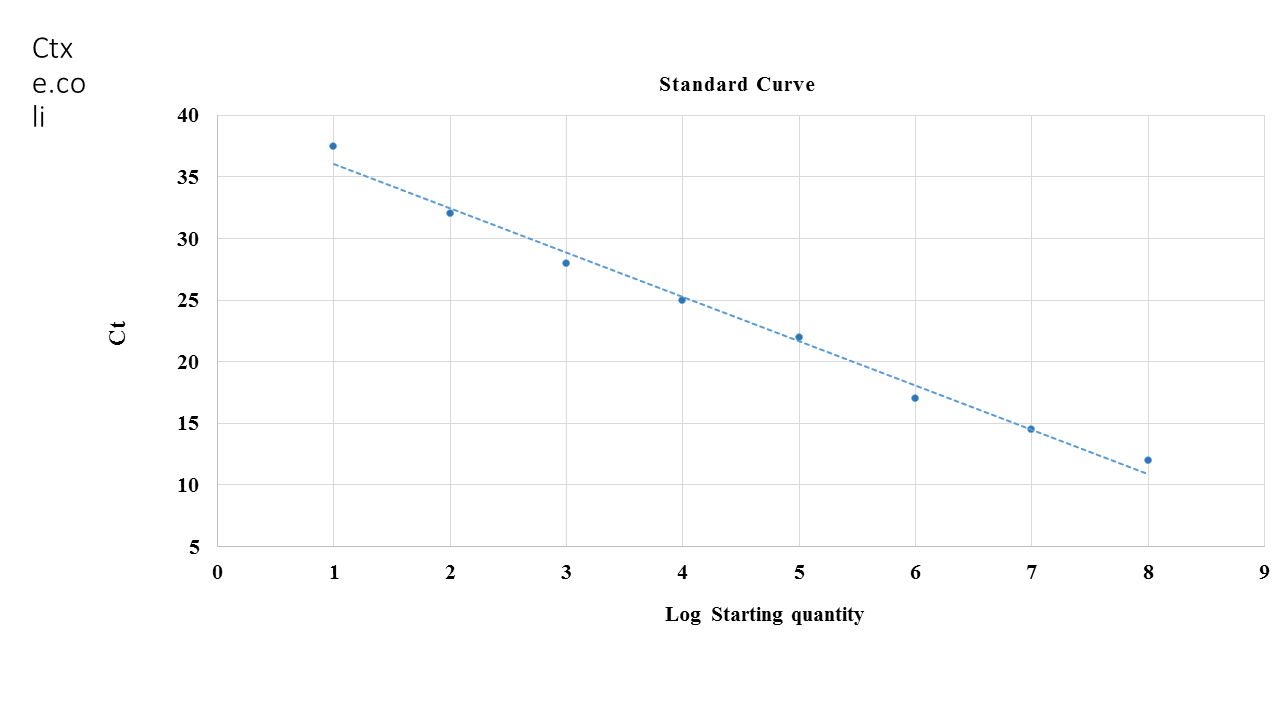


**Log gene copy number**

**SI Figure 5**: The slope S= -3.59 and efficiency E=97.19% was determined with a correlation coefficient

of R²=0.9898

***E. coli*( *yccT) bla_tem_***


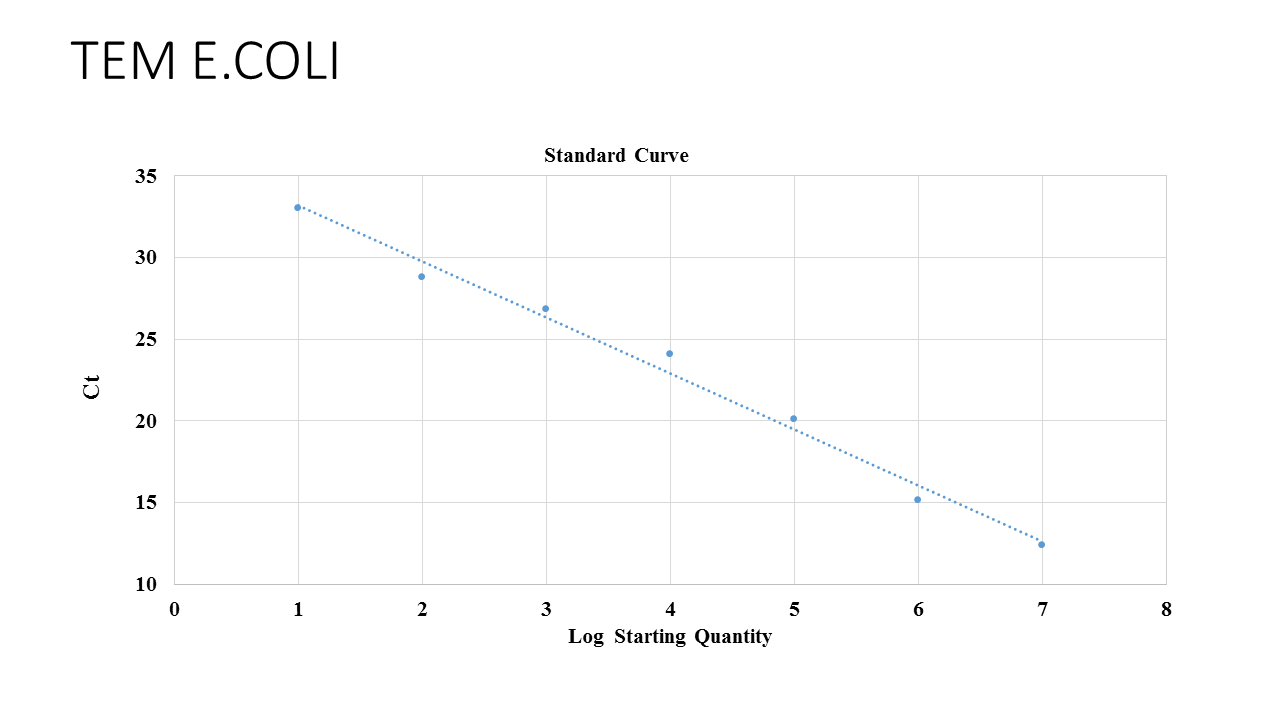


**Log gene copy number**

**SI Figure 6**: The slope S= -3.42 and efficiency E=96.9% was determined with a correlation coefficient

of R²=0.9886

### *Pseudomonas aeruginosa ( ecfX ) bla_vim_*

**
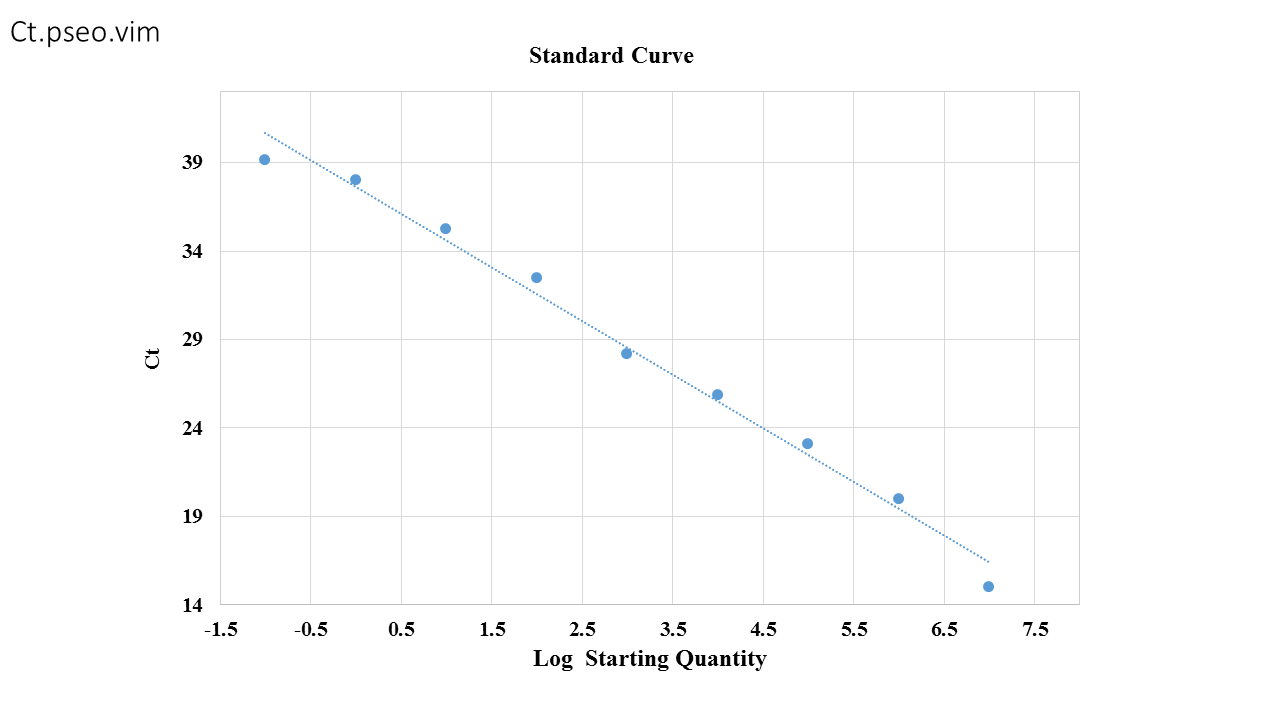
**

**Log gene copy number**

**SI Figure 7**: The slope S= -3.38 and efficiency E=97.2% was determined with a correlation coefficient

of R²=0.992

**SI Table 2: Ozone utilization efficiency (OUE) in ozonation of hospital wastewater**

| **Ozonation** | | | | | |
| --- | --- | --- | --- | --- | --- |
| **[O3]I (mg)** | **[O3]O (mg)** | **Cin (mg/l)** | **Cout (mg/l)** | **[O3]D (mg)** | **OUE (%)** |
| 4.80 | 0.04 | 22.56 | 0.96 | 0.90 | 80.42 |
| 9.60 | 0.15 | 22.56 | 1.92 | 1.9 | 78.65 |
| 14.40 | 0.35 | 22.56 | 2.88 | 2.10 | 82.99 |
| 19.20 | 0.61 | 22.56 | 3.83 | 2.92 | 81.61 |
| 24.00 | 0.96 | 22.56 | 4.79 | 3.90 | 79.75 |
| 28.80 | 1.38 | 22.56 | 5.75 | 4.05 | 81.15 |
| 33.60 | 1.88 | 22.56 | 6.71 | 4.20 | 81.90 |
| 38.40 | 2.45 | 22.56 | 7.67 | 5.04 | 80.49 |
| 43.20 | 3.11 | 22.56 | 8.63 | 6.14 | 78.59 |
| 48.00 | 3.83 | 22.56 | 9.59 | 7.57 | 76.25 |

1. Leylabadlo, H.E.*, et al.* Gut microbiota in nonalcoholic fatty liver diseases with and without type-2 diabetes mellitus.. *European Journal of Gastroenterology & Hepatology* (20201).

2. Deccache, Y.*, et al.* A qPCR and multiplex pyrosequencing assay combined with automated data processing for rapid and unambiguous detection of ESBL‑producers Enterobacteriaceae. *AMB Expr* **5**(2015).

3. Pallares-Vega, R.*, et al.* Determinants of presence and removal of antibiotic resistance genesduring WWTP treatment: A cross-sectional study. *Water Research* **161**, 319-328 (2019).

4. Rodriguez-Mozaz, S.*, et al.* Occurrence of antibiotics and antibiotic resistance genes in hospital and urban wastewaters and their impact on the receiving river. *Water Research* **69**, 234-242 (2015).

5. Aruhomukama, D.*, et al.* blaVIM- and blaOXA-mediated carbapenem resistance among Acinetobacter baumannii and Pseudomonas aeruginosa isolates from the Mulago hospital intensive care unit in Kampala, Uganda. *BMC Infectious Diseases* **19**(2019).

6. Awad, Y.*, et al.* Monitoring Antibiotic Residues and Corresponding Antibiotic Resistance Genes in an Agroecosystem. *Chemistry* **2015**(2015).

7. Calero-Cáceres, W.*, et al.* Sludge as a potential important source of antibiotic resistance genes in both the 3 bacterial and bacteriophage fractions. *Environmental Science & Technology* (2014).

8. Hembach, N.*, et al.* Occurrence of the mcr-1 Colistin Resistance Gene and other Clinically Relevant Antibiotic Resistance Genes in Microbial Populations at Different Municipal Wastewater Treatment Plants in Germany. *Frontiers in Microbiology* **8**(2017).

References:
